# Small intestinal model for electrically propelled capsule endoscopy

**DOI:** 10.1186/1475-925X-10-108

**Published:** 2011-12-16

**Authors:** Sang Hyo Woo, Tae Wan Kim, Zia Mohy-Ud-Din, Il Young Park, Jin-Ho Cho

**Affiliations:** 1 Department of Elec. Eng. and Computer Science, Kyungpook National Univ., Daegu, South Korea; 2 Department of Physiology, College of Veterinary Medicine, Kyungpook National Univ. Daegu, South Korea; 3 Department of Medical and Biological Engineering, Kyungpook National Univ. Hospital, Daegu, South Korea; 4 Department of Biomedical Engineering, Dankook Univ., Cheonan, South Korea

## Abstract

The aim of this research is to propose a small intestine model for electrically propelled capsule endoscopy. The electrical stimulus can cause contraction of the small intestine and propel the capsule along the lumen. The proposed model considered the drag and friction from the small intestine using a thin walled model and Stokes' drag equation. Further, contraction force from the small intestine was modeled by using regression analysis. From the proposed model, the acceleration and velocity of various exterior shapes of capsule were calculated, and two exterior shapes of capsules were proposed based on the internal volume of the capsules. The proposed capsules were fabricated and animal experiments were conducted. One of the proposed capsules showed an average (SD) velocity in forward direction of 2.91 ± 0.99 mm/s and 2.23 ± 0.78 mm/s in the backward direction, which was 5.2 times faster than that obtained in previous research. The proposed model can predict locomotion of the capsule based on various exterior shapes of the capsule.

## 1. Introduction

With changes in social life patterns and stress levels, the incidences of digestive diseases are increasing every year. One of the conventional diagnosis methods for digestive diseases is endoscopy, which causes pain and discomfort to patients. In addition, an endoscopy cannot easily monitor the small intestine because it is difficult to insert the endoscope through the pylorus. In order to solve these problems, many biomedical devices have been developed and the capsule endoscopy is one of the successful devices that can automatically capture internal images of the gastrointestinal tract [1-5]. One disadvantage of the capsule endoscope is its lack of locomotive ability; the capsule naturally goes in an aboral direction by peristalsis and there is no way to go backward and get detailed images when the capsule passes a suspicious position.

In order to solve this problem, many studies have been carried out to implement self-propelled robotic capsule endoscopes using various mechanisms such as motors [6-9], shape memory alloys (SMA) [10,11], magnets [12-14], and electrical stimuli [15-17]. The motor and the SMA were found to provide enough force to propel the capsule and can work together with various types of conventional gears and wheels. One disadvantage of those techniques was the large power consumption and the inability to operate from conventional batteries in the capsule endoscope. Therefore, this method was tested with power lines and the site of operation was limited to the large intestine.

Another mechanism used magnetic power to propel the capsule. M. Sendoh *et al*. implemented an exterior capsule with a screw shape and applied the external magnet to spin the capsule [12]. During the spinning, the screw shape generated forward or backward force to move the capsule. One disadvantage of this mechanism was the meandering path of the small intestine. In order to propel the capsule, the external magnetic force had to be perpendicularly applied to the direction of the movement and it was difficult to apply the magnetic force as the path of the small intestine twisted in various directions.

Another mechanism used an electrical stimulus to propel the capsule [15-17]. Park *et al*. implemented a capsule with a practical size that can propel itself in an aboral direction; Moon *et al*. reported that the capsule can propelled in both aboral and oral directions using four electrodes. This mechanism did not require complex circuits and consumed less power that make possible to operate by small batteries. Previous researches showed the feasibility of using electrical stimulus for locomotion, but did not indicated the optimal exterior shape and position of the electrodes because there was no proper model for locomotion.

Many studies have been conducted to determine the physiological properties of the small intestinal tract using a sodium channel [18], wave equation [19-21], nonlinear equation [22], and neural network [23]. These methods were focused on determining the electrophysiology that is the control signal of the peristalses. Therefore, those models did not provide basic information about the friction, contraction forces, and viscoelastic properties of the small intestine. Another method was assuming the small intestine as a viscous fluid and using fluid engineering [24,25]. This model did not consider the elastic properties of the small intestine and the values of the friction were lower than in the actual intestinal environment. Still another method used biomechanical modeling of the small intestine for robotic endoscopy [26]. The model was focused on finding an ischemia problem occurring in the small intestine when it was excessively extended by the robotic endoscope. Therefore, this model also did not report information on friction and contraction forces induced by electrical stimulation.

In this paper, a small intestinal model for an electrically propelled capsule was proposed and verified from *in vitro *experiments. The model took into account friction and the contraction force properties of the small intestine, and then conducted a simulation to choose the proper exterior shape of the capsule. Through the simulation, two shapes of capsules had chosen based on the velocity and internal volume of the capsule. After implementation of the chosen capsules, there were inserted into fresh small intestines that were temporally reactivated, and then the velocity of the moving capsule was repeatedly measured while applying electrical stimuli. From the experiments, the velocities of the capsules showed a similar tendency to the simulation results.

## 2. Method

### 2.1 Mechanism of the electrically propelled capsule

**Figure 1:**
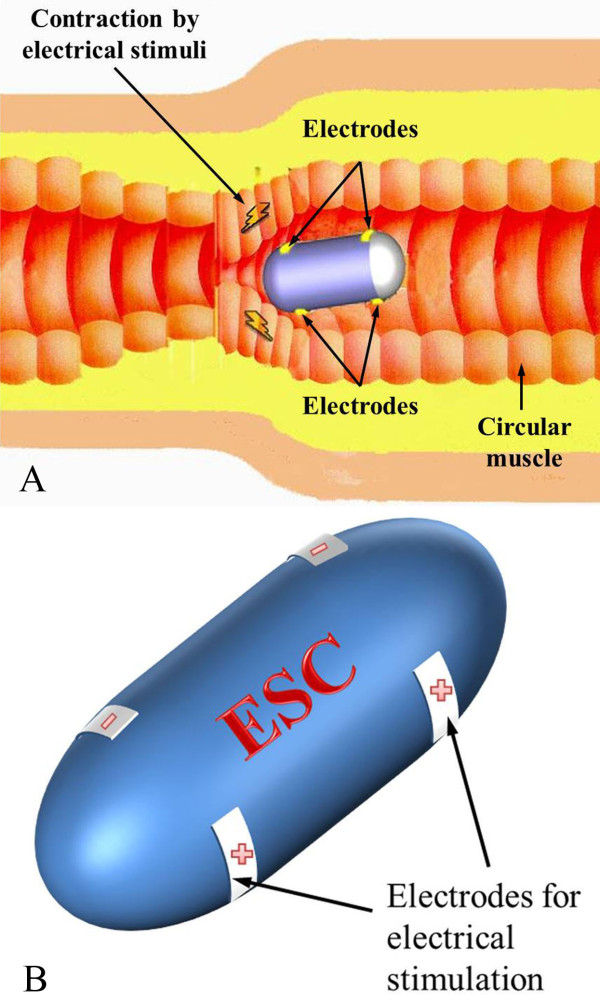
**Concept of the electrically propelled capsule**. (a) Illustration of electrical stimulus capsule (ESC) moving toward to right. (b) Shape of the ESC.

The shape of the capsule and the mechanism of the electrical stimulus capsule are illustrated in Figure 1, which shows how the smooth muscle is contracted by the electrical stimuli that is applied by a pair of electrodes and the capsule is propelled along the lumen.

**Figure 2:**
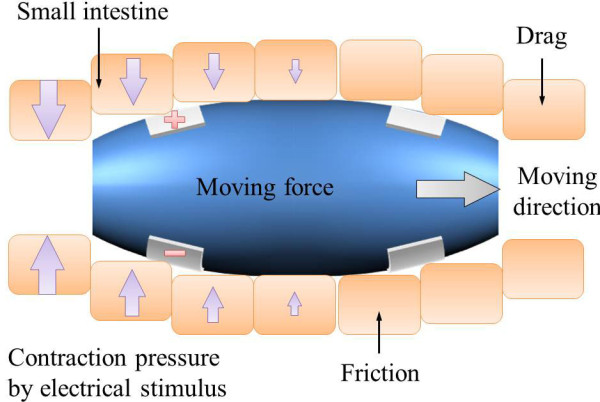
**Major forces for locomotion of the capsule**.

In order to move the capsule, the contraction force of the small intestine causes it to move the capsule, as shown in Figure 2. It is assumed that the capsule is moving to the right and small intestine is contracted by electrical stimuli. Since the capsule is moving toward the right, the left part of the small intestine is stimulated for a long time and causes large contraction force. The small intestine is composed of smooth muscle and it requires long time to fully contract compared with the skeletal muscle. The middle part of the small intestine is just entered stimulus region and contraction force is lower than that of the left part. Since the contraction force is perpendicular to the direction of movement, the exterior shape of the capsule determines the moving speed. In addition, the friction is proportional to the contraction force and the drag is dependent on the viscosity of the small intestine and velocity of the capsule. The relationship between the three forces and total moving force can be described as

(1) $$\vec{F_{t}}=\vec{F_{m}}-\vec{F_{f}}-\vec{F_{d}}$$

where ${\vec{F}}_{t}$ is the total moving force, ${\vec{F}}_{m}$ is the moving force ${\vec{F}}_{f}$ is friction, and ${\vec{F}}_{d}$ is the drag force.

**Figure 3:**
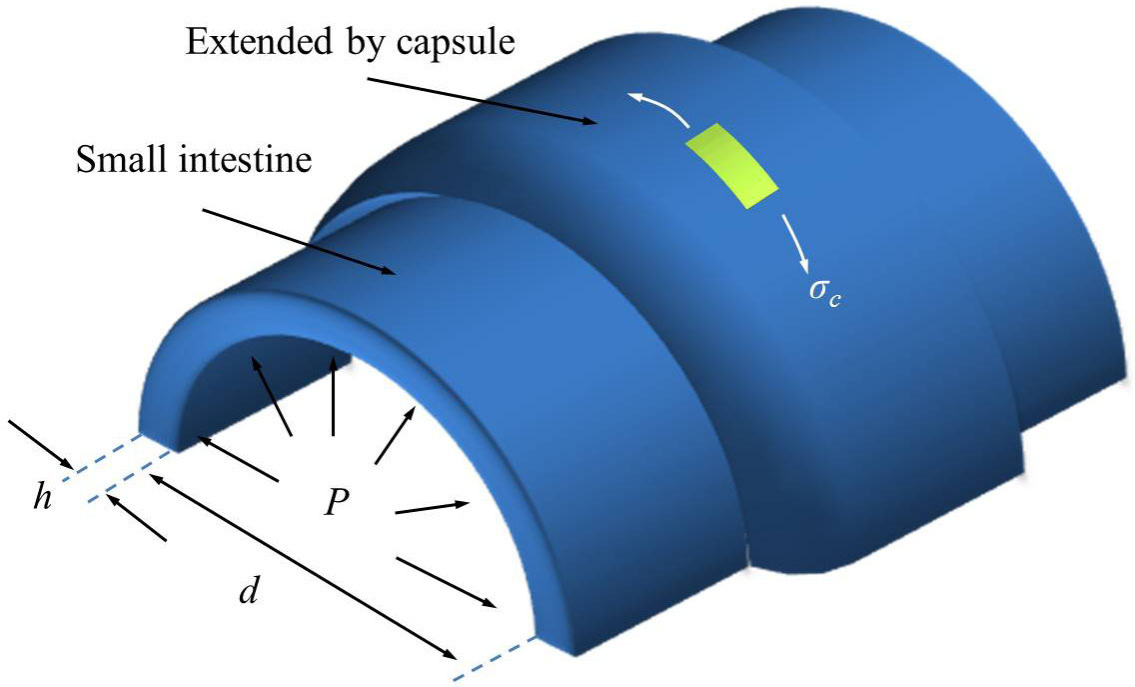
**Illustration of simplified small intestine model when capsule is in the intestine**.

Figure 3 illustrates how the intestine is extended like a thin cylindrical vessel when the capsule is in the small intestine and how it applies strain to the small intestine. Since the small intestine has viscoelastic properties, the internal pressure slowly decreases as time passes. The stress value of the small intestine was measured by Baek *et al*. [27] and the stress was found to be

(2) $$\sigma_{c}\left(t\right)=\varepsilon_{c}\left(0.7e^{-\frac{t}{18}}+0.63e^{-\frac{t}{1.6}}+0.92\right)$$

(3) $$\varepsilon_{c}=\frac{f\left(x\right)}{d_{0}-1},\;\varepsilon_{c}>0$$

where *ε*_c _is strain, *t *is time, *f *(*x*) is the exterior shape of the capsule, and *d*_0 _is the initial diameter of the small intestine. Since the capsule extends the small intestine, the strain depends on the exterior shape of the capsule. The exterior shape of the capsule will be discussed later.

The small intestine has similar shape of the thin cylindrical vessel and internal pressure of the thin cylin-drical vessel can be express as

(4) $$P_{i}=\frac{2h}{d}\sigma_{c}$$

where *d *is diameter of the small intestine and *h *is the thickness of the small intestine. Therefore, internal pressure of the small intestine can be express as formula 5 and the pressure is changed by time, diameter of the capsule, and the exterior shape of the capsule.

(5) $$P_{i}=\frac{2h}{d}\;\left(\frac{f\left(x\right)}{d_{0}}-1\right)\left(7e^{-\frac{t}{18}}+6.3e^{-\frac{t}{1.6}}+5\right)$$

**Figure 4:**
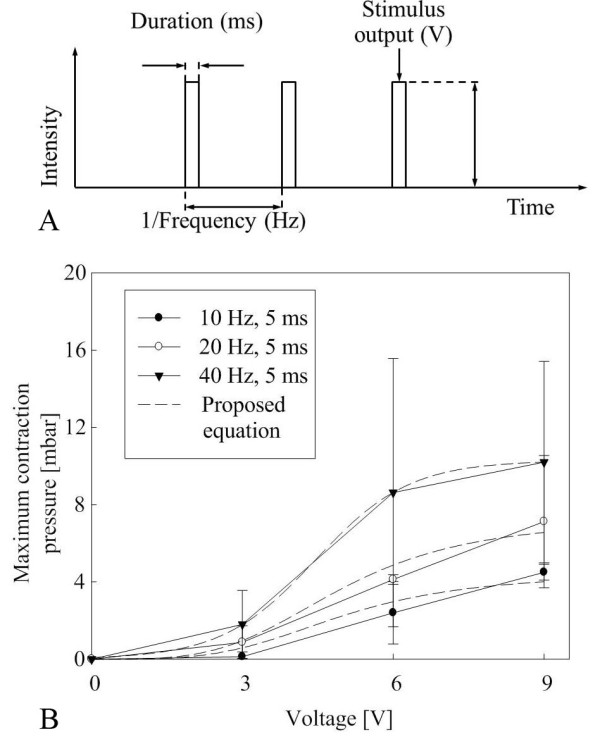
**Contraction experiment results**. (a) Electrical stimulus parameters. (b) Maximum contraction pressure.

When an electrical stimulus is applied to the small intestine, a high contraction force is generated, de-pending on the time and electrical stimulus parameters. In previous experiments, the maximum contraction force and rising time constant depended on the electrical stimuli were measured using square electrodes (5 × 6 mm) with a catheter and Figure 4 summarizes these results [28,29]. Detail experimental processes are included in the additional file 1.

Figure 4 (a) depicts the electrical stimulus parameters, Figure 4 (b) shows the maximum contraction pressure depending on various electrical stimulus parameters, and Figure 4 (c) shows transient response of contraction. In order to reduce the number of experiments, the duration was fixed at 5 ms that is twice of chronaxie of the smooth muscle. The experimental results shows that the maximum contraction force increased nonlinearly when the voltage was increased.

From the experimental results, the maximum contraction pressure is modeled by using logistic regression and it is shown as

(6) $$P_{m}=\frac{0.24f_{s}+1.18}{1+{\left(\frac{A_{s}}{4.97}\right)}^{-3.72}}$$

where *P*_m _is the maximum contraction pressure, *f*_s _is the stimulus frequency, and *A*_s _is the stimulus amplitude. Standard coefficient error is lower than 0.2 and the P-value showed less than the significance level (< 0.01).

After decision of the maximum contraction pressure, transient response of the contraction is modeled as simple first-order ordinary differential equation and it is shown as

(7) $$\frac{dP_{s}\left(t\right)}{dt}+\frac{1}{\tau_{s}}P_{s}\left(t\right)=\frac{P_{m}}{\tau_{s}},\;P_{s}\left(0\right)=0$$

where *P_s _*(*t*) is the contract pressure, *τ*_s _is the rising time constant, *P*_m _is the maximum contraction force.

The value of the rising time (*τ*_s_), which is defined as a time to reach 63.2% of its maximum contraction pressure, was measured from for three different stimulus parameters (10, 20, and 40 Hz @ 6 V and 5 ms) with four different samples from two different swains (N = 20). The average (SD) rising time constant value was measured as 17.3 ± 8.3 seconds.

It is assumed as the pressure is locally distributed in Gaussian form because the pressure was highly generated from the small electrode, and the size of the balloon catheter was large enough to measure the averaged pressure data. The Gaussian equation is shown as

(8) $$G\left(x\right)=1.4e^{-\frac{{\left(x\;-\;x_{0}\right)}^{2}}{2\omega }}$$

where *x *is the *x-axis, x_0_*is the center placement of the electrode, and *ω *is the variance. The variance value was empirically set as 0.22.

**Figure 5:**
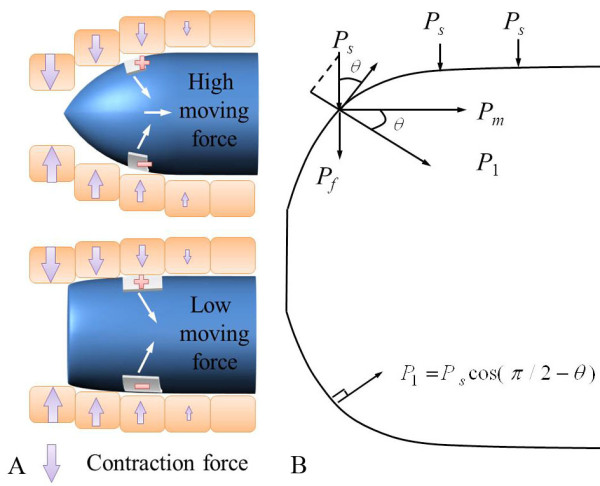
**Capsule shape and moving speed**. (a) Illustration of faster velocity of streamlined capsule compared to cylindrical capsule. (b) Mathematical relationship between the shape of the capsule and moving force.

Figure 5 (a) illustrates the importance of the exterior shape and it shows that the streamlined shape is better than the cylindrical shape. Figure 5 (b) summarizes the relationship between the contraction force and the shape of the exterior of the capsule.

The exterior shape of the capsule is modeled using the exponential function as

(9) $$f\left(x\right)=\left\{\begin{array}{cc} \left(R-r\right)\left(1-e^{-\alpha x}\right)+r, & 0\leq x<\frac{L}{2}\\ \left(R-r\right)\left(1-e^{\alpha \left(x-L\right)}\right)+r & \frac{L}{2}\leq x\leq L \end{array}\right)$$

where *r *and *R *are the initial and maximum radii, *α *is the dimensionless constant that determines the slope of the capsule, and *L *is the length of the capsule. Figure 5 (b) depicts the relationship between the contraction force and moving force. The moving pressure can be described as

(10) $$P_{m}=\left\{\begin{array}{c} P_{1}\cos\left(\theta \left(x\right)\right),0\leq x<\frac{L}{2}\\ -P_{1}\cos\left(\theta \left(x\right)\right),\frac{L}{2}\leq x\leq L \end{array}\right)$$

(11) $$P_{1}=\left(P_{s}+P_{i}\right)\cos\left(\frac{\pi }{2}-\theta \left(x\right)\right)$$

(12) $$\theta \left(x\right)=\frac{\pi }{2}-\tan^{-1}\left(f^{\prime }\left(x\right)\right)$$

Where *P_s_*and *P_i_*are the contraction pressure by the electrical stimulus and internal pressure from extension by the capsule, and *θ*(*x*) is the slope of exterior capsule. The force can be calculated from the distribution of pressure to force when integrated over the exterior area of the capsule. Therefore, the moving force can summarized as formula 13.

(13) $$F_{m}=2\pi \;\int_{0}^{L}P_{m}f\left(x\right)\sqrt{1+f^{\prime }{\left(x\right)}^{2}}dx$$

In addition, the friction can be described similarly

(14) $$F_{f}=2\pi \mu \int_{0}^{L}P_{f}f\left(x\right)\sqrt{1+f^{\prime }{\left(x\right)}^{2}}dx$$

(15) $$P_{f}=P_{1}\sin\left(\theta \right)$$

Where μ is the frictional coefficient and it is set at 0.1 based on Baek's experiments [27]. The friction increases with increasing contraction force and grooves at the capsule surface.

**Figure 6:**
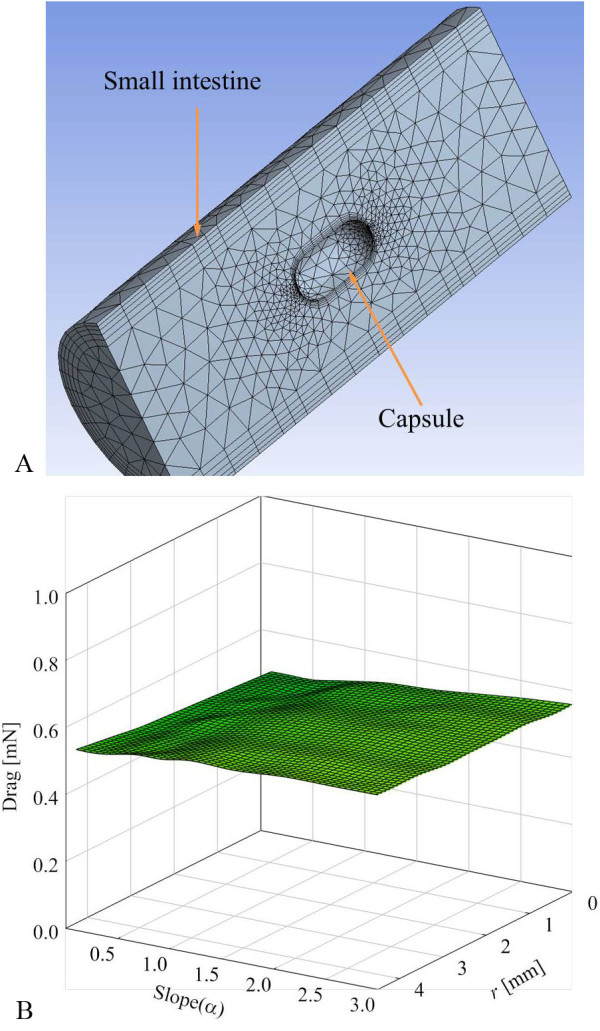
**Drag depends on the shape of the capsule**. (a) Illustration of finite simulation with meshes. (b) Drag depends on various shapes of the capsule at fixed velocity (0.5 mm/s).

Another force is drag, which is highly influenced by the viscosity properties of the small intestine. Since the moving speed of the capsule is very slow, it satisfies Stokes' fluid law. The Stokes' drag is

(16) $$F_{d}=b\;v$$

where *b *is the drag coefficient and *v *is the velocity of the capsule. In order to calculate the drag coefficient of various shapes of the capsule, a computational fluid dynamics program (CFD, ANSYS) is used and the Navier-Stokes equation is calculated. For the simulation, the small intestine is assumed to be a non-compressible viscous liquid [30], laminar flow, Newtonian fluid, steady state, and no slip condition. Figure 6 (a) shows 9512 generated meshes and Figure 6 (b) shows that the drag depends on the varying slope and initial radius when the velocity of moving capsule is 0.5 mm/s. When the moving speed is slow, the exterior shape of the capsule does not highly affect.

**Figure 7:**
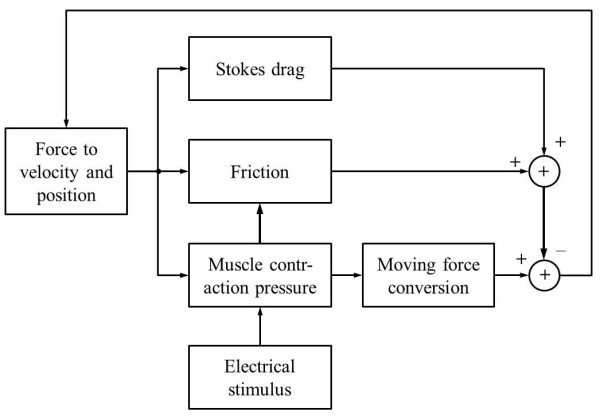
**Block diagram of simulation**.

Figure 7 depicts a block diagram for the calculation of the above equations. When the electrical stimulus is applied, the drag, friction, and moving force are calculated using the velocity and position information of the capsule. The friction, drag, and moving force were recalculated every 0.001 second to prevent a diversion problem.

## 3. Simulation and experimental results

**Figure 8:**
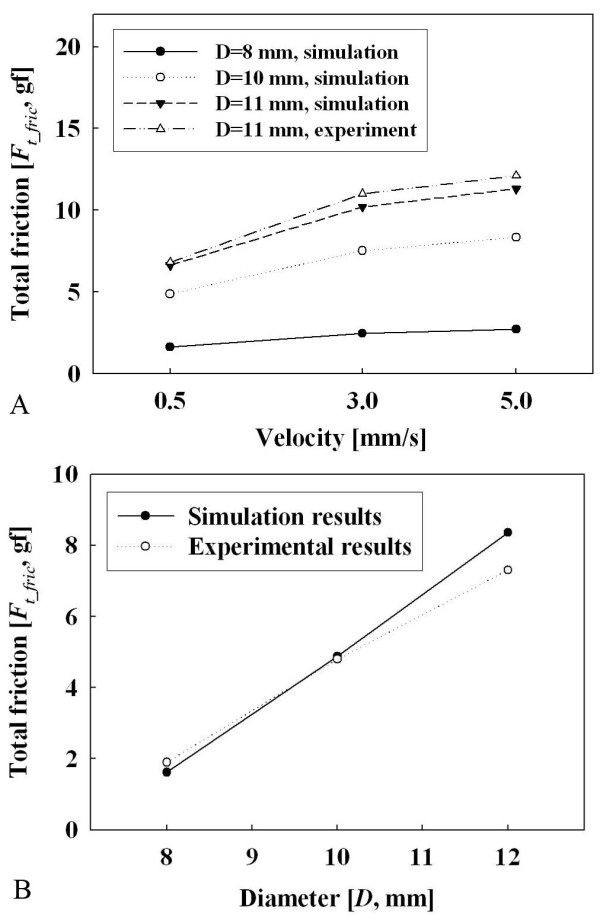
**Comparison between Wang's experiments **[31]**and the our simulation results**. (a) Relationship between friction and diameter. (b) Relationship between velocity and friction.

In order to verify the simulation, the result was compared with Wang's experiments [31], which measured the friction of capsules of various shapes, and it is shown in Figure 8. Figure 8 (a) shows that the friction is greatly affected by the diameter of the capsule and Figure 8 (b) demonstrates that the friction is affected by the velocity of the capsule. Wang *et al*. tested a fixed diameter with various velocities and our proposed simulation model showed similar tendency dependent on the velocity and diameter of the capsule.

**Figure 9:**
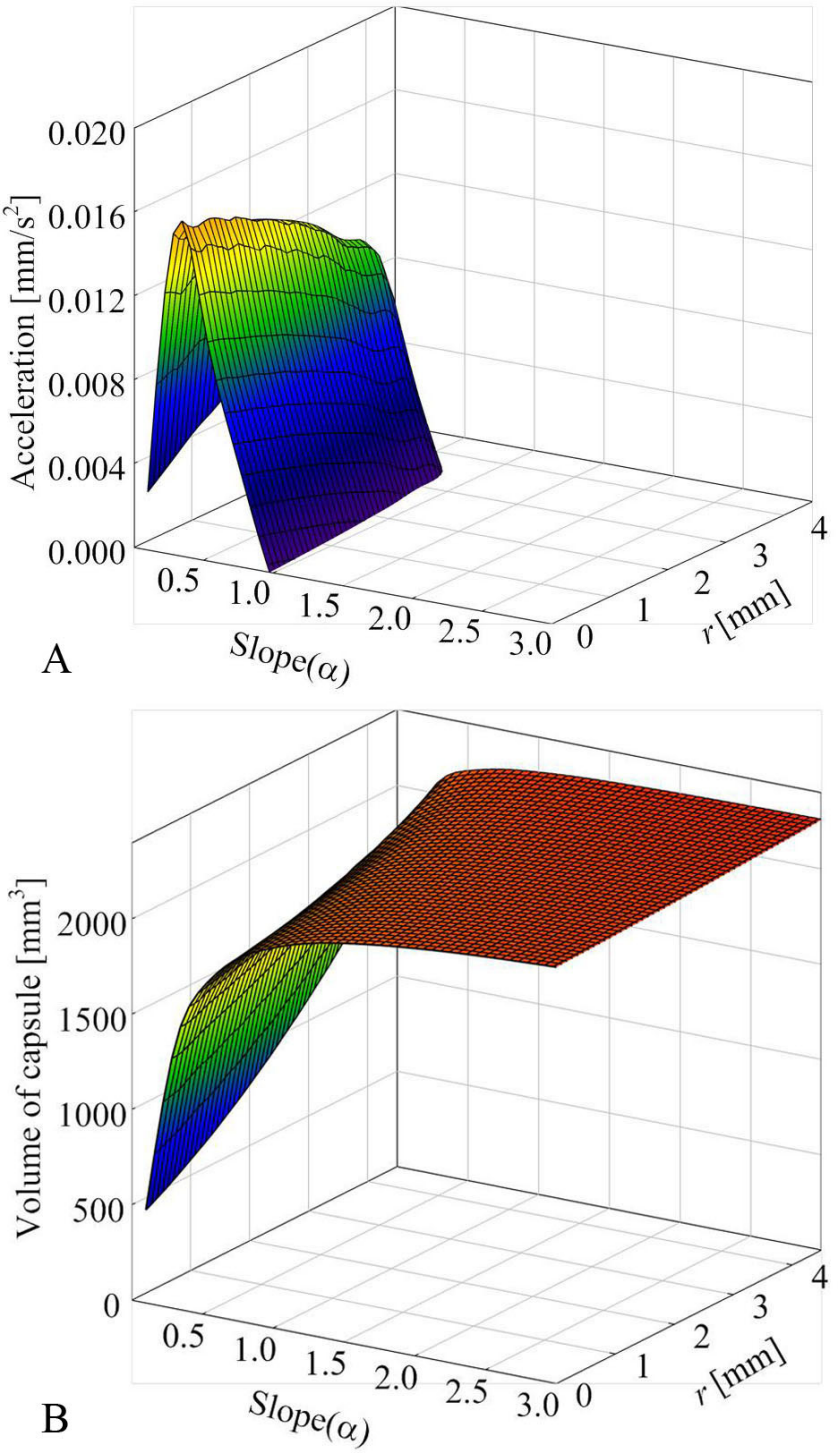
**Exterior shape simulation results**. (a) Relationship between acceleration and shape of the capsule. (b) Relationship between volume and shape of the capsule.

Wang *et al*. did not measure the friction difference between various types of electrical stimuli. In our study, two electrically stimulated capsules were implemented based on our simulation results and one another capsule was implemented for control group. In order to reduce number of animal experiments, the maximum diameter was fixed at 11 mm, which is normal for a telemetry capsule and the same diameter that Wang et al. used. Although it is true that the maximum diameter plays a major role in the friction, the diameter of the capsule cannot easily reduced due to the problem of battery capacity. Figure 9 shows that simulation results depend on the slope and initial diameter of the capsule. The results show that the streamlined capsule is faster than the cylindrical capsule, but the streamlined capsule is difficult to fabri-cate.

**Figure 10:**
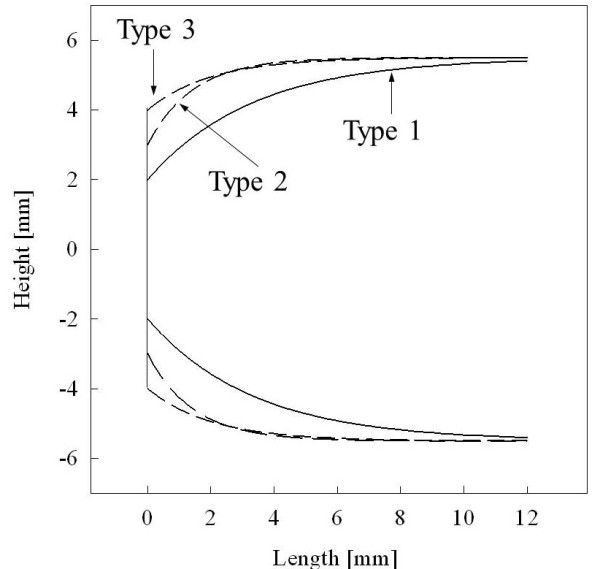
**Cross sectional view of three types of capsules**.

In order to determine the proper shape for the capsule, the acceleration and volume were compared and convergence points were chosen. Since the streamlined capsule has a very small internal volume, it is a great challenge for the engineer to put circuits into the capsule. Additionally, the inside volume requirement for telemetry capsules varies for its application; therefore, the optimal shape of the capsule depends on how large an inside volume is required. Since the requirement volume varies for each application, two types of capsules were suggested, both of which can have the proper inside volume while being able to move at a high enough speed. These values are just guidelines for other engineers and a value could be chosen within the range presented depending on the application. Figure 10 shows three types of capsules. Type 2 (*r*: 2 mm, *τ*: 0.3) has a larger internal volume than type 1 (*r*: 3 mm, *τ*: 0.3) but is 23% slower than the type 1 capsule. Type 3 (r: 4 mm, τ: 0.3) was chosen as a control group, and it has a slightly larger inside volume than type 2 but it did not move at all from the simulation.

**Figure 11:**
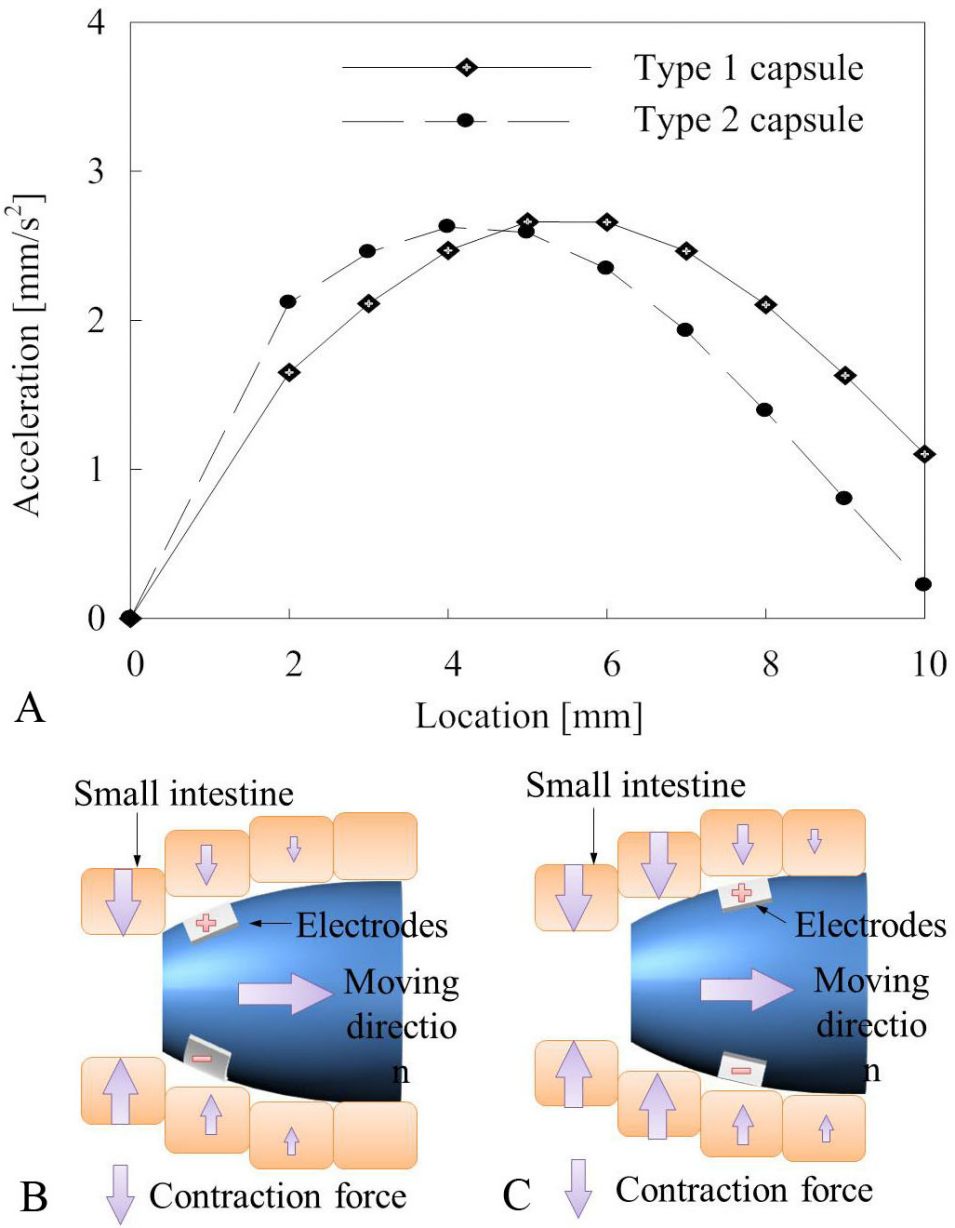
**Electrode position simulation**. (a) Relationship between location and acceleration. (b) Illustration of moving capsule when electrodes are placed at the left edge of the capsule. (c) Illustration of moving capsule when electrodes are located a bit to the right of the left edge of the capsule.

After simulation of the shapes of the capsules, another simulation is conducted to find the proper location for the electrodes. Figure 11 shows simulation results and location 0 indicates that the electrodes are at the left edge of the capsule. From the simulation, we found that it is better to place the electrodes about 5 mm from the left edge of the capsule depending on its shape. This is because of a contraction time delay from the small intestine, and this is illustrated in Figure 11 (b) and 11(c) while the size of arrow represents the centration force from the small intestine. When the electrodes are placed at the edge of the capsule, the right part of the small intestine is just entered stimulating region and the small intestine does not produce a significant enough contraction force. When the electrode is placed 5 mm from edge, the right part of small intestine is pre-contracted and can produce more force when the capsule moves to the right. When the elec-trodes are placed more than 5 mm from the edge, the friction is increased and eventually decreases the acceleration.

**Figure 12:**
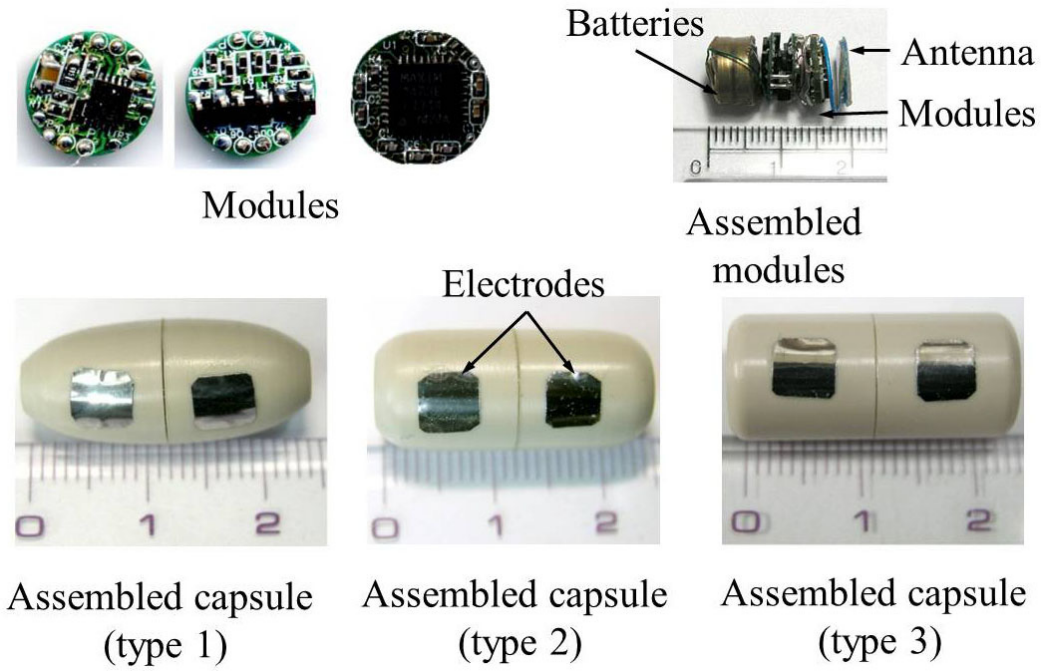
**Modules, assembled modules, and final capsule types**.

**Figure 13:**
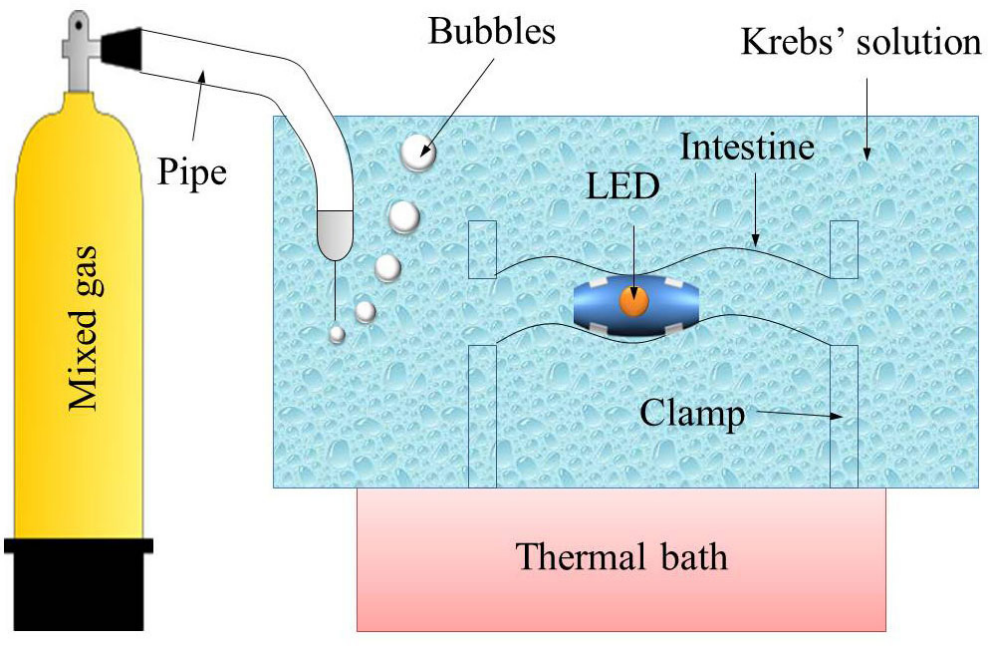
**Illustration of experimental setup**.

Figure 12 shows the implemented modules, assembled modules, and three different types of exterior capsules. Implementation of the capsule has been well described in previous studies [16,32], and it is controlled by wireless transmitter. Figure 13 illustrates the experimental setup. Mixed gas (95% O_2 _and 5% CO_2_) was continually injected into the Krebs' solution to maintain its pH.

Six Landrace porcine small intestines (6~7 months old) were collected from a local abattoir. The small intestines were rinsed several times and transported to the laboratory in ice cold oxygenated Krebs-Ringer bicarbonate solution. This experiment was performed according to the guidelines of the Committee on Animal Experimentation of Kyungpook National University. Six different small intestines were taken and inserted into the Krebs' solution to await activation [28]. After the small intestine is activated, it naturally performs peristalsis, secretes digestive juice, and can be contracted by electrical stimuli.

**Figure 14:**
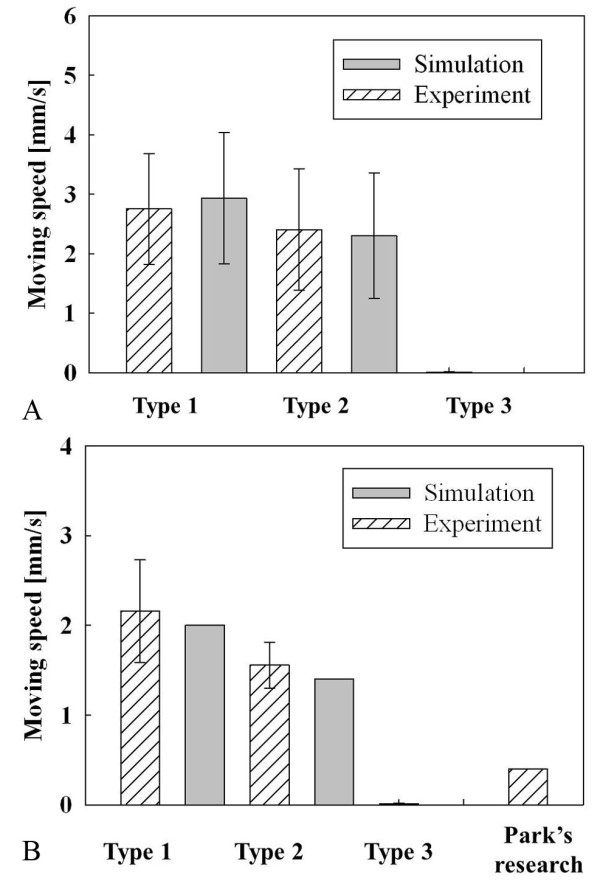
***In vitro *experimental results**. (a) Relationship between type of capsule and moving speed (n = 72). (b) Velocity comparison with previous research (n = 24).

Figure 14 shows a summary of the experimental results. In order to reduce the number of experiments, the electrical stimulus parameters such as voltage and duration were fixed at 6 V and 5 ms, and the frequency was changed as 10, 20, and 40 Hz. The total of 72 experiments were conducted for the three types of capsules, and the type 1 capsule was found to be faster than the type 2 capsule (P < 0.05, t-test). Since the frequency is changed, the simulation results also have a standard deviation. In order to reduce the muscle fatigue problem, 3 minutes interval was taken between electrical stimuli. Figure 14 (b) shows the experimental results compared with a previous electrical stimulus capsule [15] and the proposed capsules. Previous research used a relatively long duration and fixed frequency (6 V, 10 Hz, 50 ms), and the most similar stimulation results from current experiment (6 V, 10 Hz, 5 ms) data were compared. Even though the total energy applied to the small intestine was 10 times lower than previous studies, the average moving speed of current experiments was 5.2 times faster than the previous research.

**Figure 15:**
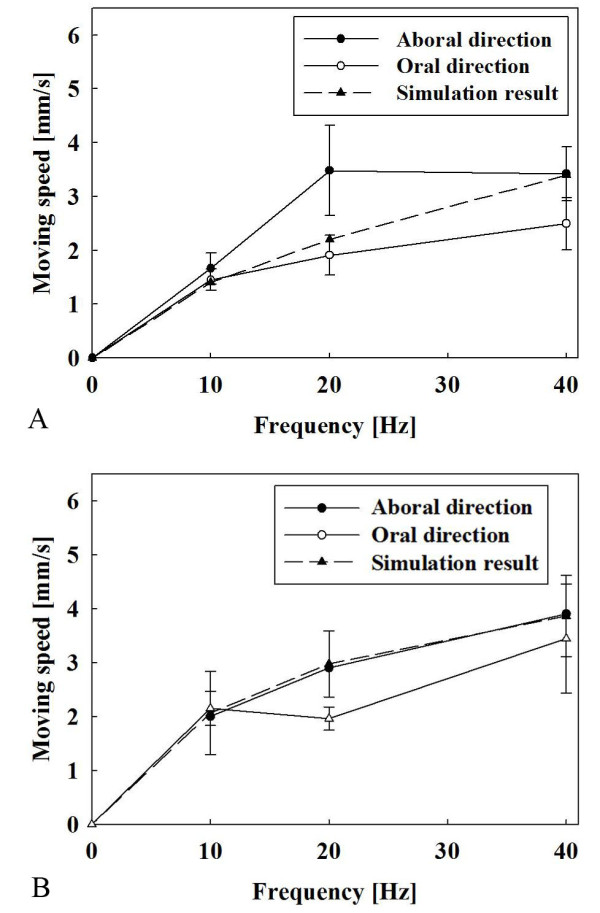
**Relationship between frequency and moving speed**. (a) Type 1 capsule. (b) Type 2 capsule.

Figure 15 shows detailed results of the experiments, especially the relation between various frequencies and moving speeds. Both moving speed of the capsules are proportional to the frequency, and there is a significant difference between the aboral and oral direction moving speeds (Mann-Whitney rank sum test, P < 0.05). Since the moving speed of the capsule is proportional to the frequency, the frequency parameter can be used to control the moving speed of the capsule.

## 4. Conclusion and discussion

In this paper, a simulation model for an electrical stimulus capsule was proposed and compared with ex-perimental results. The model represented friction and drag related to the shape of the capsule and the contraction force from electrical stimuli. From the model, the acceleration and velocity of the capsule were calculated. The simulation model was compared with the experimental results and the two were found to be well matched.

After the electrical stimulus, there were no visual signs of bleeding or obstruction and the capsule moved freely to the oral and aboral direction in the small intestine.

One interesting finding was that after the electrical stimulus was completed, sometimes-large contractions occurred and moved the capsule. In this experiment, movement after stopping the capsule was not included, but was sometimes observed in certain positions of the small intestine. This phenomenon was not observed at every points of small intestine, but it was reproducible at certain points where this phenomenon was observed. This could interfere with stopping the capsule at certain positions and additional breaking mechanisms for the capsule should be researched [33,34].

Another concern was the distorted image quality when the capsule was moving fast in the small intestine. An additional file 2 shows a movie that was captured at 30 frames per second while the capsule was moving in the small intestine by using electrical stimulus. It can easily be seen in the video that small bubbles and foam were generated by the capsule's movement, blocking the image of the small intestine. It is assumed that more foam and bubbles were generated than in the *in vivo *experiments because the small intestines were submerged in the Krebs' solution. It is assumed that proper bowel preparation could reduce the foam and bubbles. Therefore, additional research is required to de-termine the optimum velocity of the capsule in order to avoid reducing the image quality.

In this study, the size of electrodes were fixed at 5 × 6 mm based on previous research data. The size of the electrodes could influence the bulk impedance and contraction area. In order to determine the optimal size and shape of the electrodes, an electrical physiological model and Gauss equation mixed model is required.

The meandering path of the small intestine will cause additional friction from the walls of the small intestine. The present simulation assumes that the small intestine is a long straight valve and so does the *in vitro *experiments, and the results of both were similar. In order to straighten the small intestine, the mesentery was cut and straightened; this caused some parts of small intestine to wrinkle and sometimes the capsule was blocked in certain positions. When the capsule was passing a meandering curve, the capsule will asymmetrically stretched a part of the small intestine. Therefore, additional research is required for asymmetrical modeling of the portions of the small intestine with a curved structure.

The current model does not represent difference in the aboral and oral moving speeds. Since the friction does not depend on the direction of movement, and the contraction force from the electrical stimulus was high enough to ignore peristalsis, there was no speed difference from the simulations in either direction. It could be assumed that the small intestine would naturally resist the capsule, going in an oral direction, and it could be explained by "law of the intestine," which indicates that the small intestine pushes its contents toward to an aboral direction. Therefore, additional research is required into the velocity difference between the movement in the aboral and oral directions.

In spite of the above problems, this paper presents a practical simulation model for an electrical stimulus capsule that will help us to design the proper shape of the capsule and positions of the electrodes. Further, this model could be used to study and to reduce the number of capsule endoscopes that do not naturally go in an oral direction.

## Authors' contributions section

SHW carried out the simulation and implementation, participated in the most of process and drafted the manuscript. TWK and ZMUD carried out animal experiments. IYP participated in the simulation, especially in fluid simulation. JHC carried gives advice of implementation, especially designing the telemetry system. All authors read and approved the final manuscript.

## Competing interests

The authors declare that they have no competing interests.

## Supplementary Material

Additional files 1 **Detail explanation of measuring the contraction force while applying the electrical stimuli**. Detail explanation of experimental setup, sequence, and analysis. Click here for file

Additional files 2 **A movie file of locomotion of various types of electrical stimulus capsules**. A movie file that shows the electrical stimulus capsule can be used to be locomotion method. Click here for file
