# Supplementary material for: Small intestinal model for electrically propelled capsule endoscopy
Source: Biomed Eng Online. 2011 Dec 16;10:108. doi: 10.1186/1475-925X-10-108 (PMC3265441; doi:10.1186/1475-925X-10-108)
Supplement: Additional files 1 — Detail explanation of measuring the contraction force while applying the electrical stimuli. Detail explanation of experimental setup, sequence, and analysis. [file 1475-925X-10-108-S1.DOCX]

1. Contraction experiment using a modified balloon catheter.

In order to measure the contraction force while applying the electrical stimulus, two electrodes (5 × 6 mm) are attached to the ordinary balloon catheter (30 cc, 20 Fr), and Figure 1 shows the implementation. The thickness of the electrode was 25 µm, which does not influence the measuring sensitivity of the catheter. The flexible wires were attached on the electrode to reduce error from the wires.

Figure 2 shows calibration data with and without the electrode while applying a small pressure. The total number of measurement was 10 and they showed reasonable linearity above 0.5 mbar. When the electrical stimulus is applied to the small intestine, average contraction pressure is more than 5 mbar, which is 10 times higher than that the non-linear range.


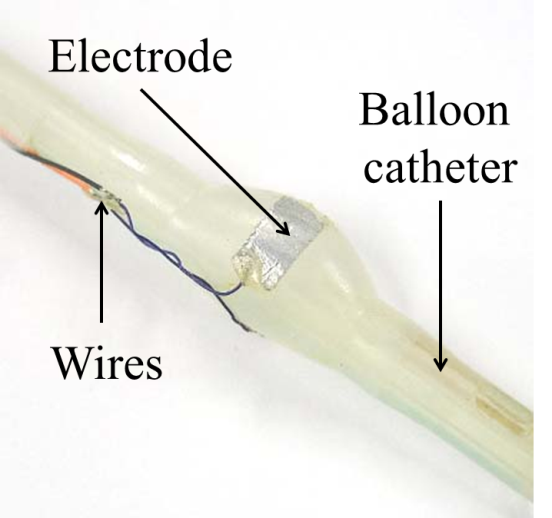


Fig. 1 Balloon catheter for measuring contraction while applying electrical stimulus.

Fig. 2 Comparison between measured pressure with and without electrodes while applying small pressure.

Figure 3 illustrates the experimental setup and the modified balloon catheter with the attached electrode is inserted into the small intestine and then the electrical stimulus is applied so that the contraction pressure can be measured. Figure 4 shows electrical stimulus parameters generated from the stimulator (Stimulator 100, UK), and contraction pressure was measured by the pressure measurement unit (MP36, Biopac system, Inc.).


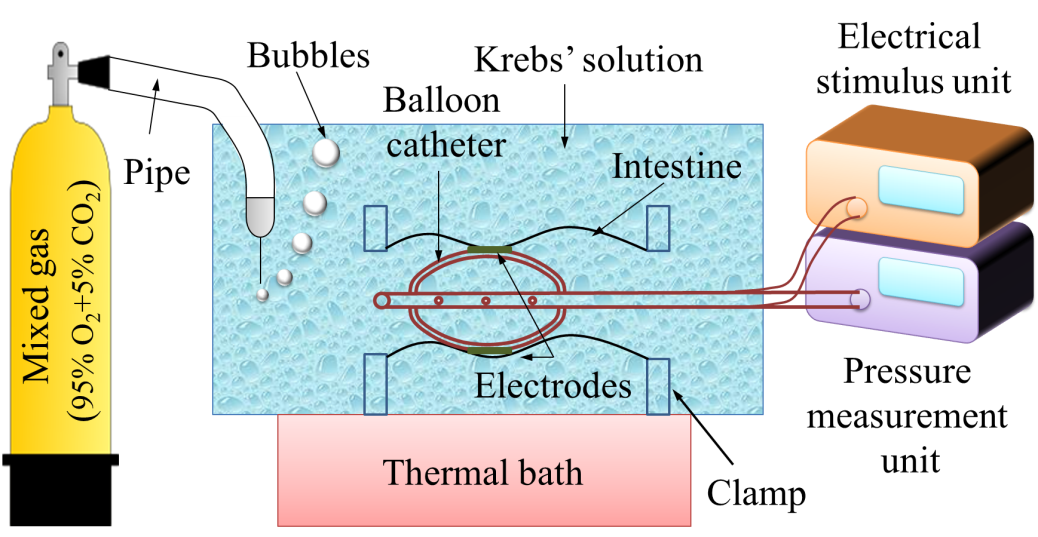


Figure 3. Experimental setup for measuring contraction pressure depending on application of the electrical stimulus.


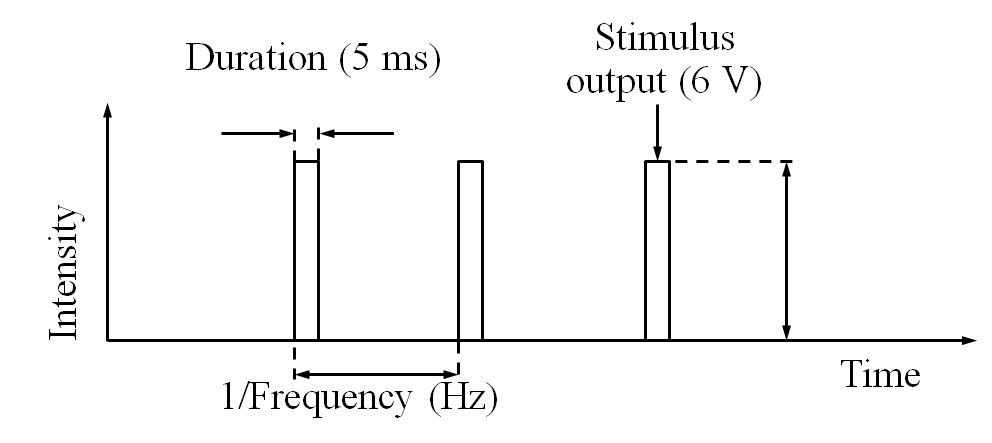


Figure 4. Electrical stimulus parameters.

The duration was fixed at 5 ms and the frequency and voltage were varied between 10, 20, and 40 Hz and 3, 6, and 9 V, respectively. A total of 45 experiments were conducted with a resting time of about 3 minutes between the electrical stimuli. In order to reduce the adaptation to electrical stimulus, the stimulus parameter was randomly applied.
